# ROS-Based Smart Walker with Fuzzy Posture Judgement and Power Assistance

**DOI:** 10.3390/s21072371

**Published:** 2021-03-29

**Authors:** Yeong-Hwa Chang, Nilima Sahoo, Jing-Yuan Chen, Shang-Yi Chuang, Hung-Wei Lin

**Affiliations:** 1 Department of Electrical Engineering, Chang Gung University, Taoyuan City 333, Taiwan; m0521042@stmail.cgu.edu.tw (N.S.); m0521018@stmail.cgu.edu.tw (J.-Y.C.); boommale@gmail.com (S.-Y.C.); 2 Department of Electrical Engineering, Ming Chi University of Technology, New Taipei City 243, Taiwan; 3 Department of Electrical Engineering, Lee-Ming Institute of Technology, New Taipei City 243, Taiwan; hwlin@mail.lit.edu.tw

**Keywords:** robot operating system (ROS), smart walker, fuzzy controller

## Abstract

In recent years the increased rate of the aging population has become more serious. With aging, the elderly sometimes inevitably faces many problems which lead to slow walking, unstable or weak limbs and even fall-related injuries. So, it is very important to develop an assistive aid device. In this study, a fuzzy controller-based smart walker with a distributed robot operating system (ROS) framework is designed to assist in independent walking. The combination of Raspberry Pi and PIC microcontroller acts as the control kernel of the proposed device. In addition, the environmental information and user postures can be recognized with the integration of sensors. The sensing data include the road slope, velocity of the walker, and user’s grip forces, etc. According to the sensing data, the fuzzy controller can produce an assistive force to make the walker moving more smoothly and safely. Apart from this, a mobile application (App) is designed that allows the user’s guardian to view the current status of the smart walker as well as to track the user’s location.

## 1. Introduction

Mobility is an important feature for each individual as it is the ability of a person to move independently. People who have mobility issues usually rely on others to do their daily routine activities. According to the report from the World Population Prospects, the number of persons aged 60 or over has increased worldwide in recent years. It is reported that the global population of aged 60 or older was 962 million in 2017 and this number is expected to double with a projected number nearly 2.1 billion by 2050 [1]. Also, from the statistics of 2018, the elderly aged 65 and over in Taiwan accounted for 14.3% of the total population, which exceeded the threshold of 14% of the United Nations definition of aging society [2]. Degenerative joint, Parkinson, and musculoskeletal deformities may be the reasons for locomotive impairment [3]. In addition, due to the deterioration of muscle strength and poor balance, there may be chances of fall-related injuries which are quite common in older adults. Thus, it is very important to develop a health care mobility aid to support the elderly for their movement or the people need to be rehabilitated. In the market, there are many types of assistive devices available to assist the elderly in their daily actions, such as canes, crutches, and conventional walkers. The cane type walker is though small in size but is a fixed structure for single-handed use. Two-handed walkers may provide better support with wide four fulcrums [4]. But necessary upper limb strength is required for such aids to be lifted up from the ground in each step to move forward. Walker with auxiliary wheels is designed for users who lack arm strength. However, the risk of falling increases while walking up or down on a ramp surface [5,6,7]. A manual brake could be added to improve operational safety, but it is not easy to use for the elderly, especially for who are weak in upper limbs. Therefore, this paper is motivated to design a smart wheel-type walker combined with peripheral sensors and fuzzy control technologies.

There are two types of walkers, passive and active. In general, the power to drive the passive walker relies entirely on the user’s strength. For examples, a passive walker is powered by the user-supplied forces with controlled brakes [8,9,10]. Recently, the walker powered by motors to steer the walker has attracted a lot of attention [11,12,13,14,15,16,17,18,19,20,21,22,23,24,25,26,27,28,29,30]. Patel et al. [11] used an active walker as the experimental platform to exploit the interactions between an intelligent mobility aid and the human operator. Shi et al. [12] developed an active walker named Walkmate, providing a negative feedback loop of the motion control with force sensors. Song et al. [13] developed a walking assistive robot named Walbot, of which a force cooperative guidance control scheme was proposed for automatic navigation. Valadao et al. [15] developed a smart walker with the detection of the user’s legs, whose distances from the laser sensor provide the necessary information to maintain safe walking. Saint-Bauzel et al. [16] proposed a fuzzy controller based robotic walker to assist patients in lower limb rehabilitation. Zhao et al. [17] proposed a walking assist robot which can detect the abnormal gait pattern in users for fall prevention. Lopes et al. [18] presented an innovative research, in which whenever a certain pressure is exerted, the walker gets slower to avoid the occurrence of falls by blocking the wheels. Kapsalyamov et al. [23] proposed a wearable robotic solution to assist the elderly for mobility. It provides the necessary force according to the predefined trajectory. Serigo et al. [24] presented a ROS-based smart walker called AGoRA walker, equipped with a sensory and actuation interface. Zhao et al. [25] proposed a robotic walker to provide a convenient-to-use indoor walking aid for the elderly. The walker supports multiple modes of interaction and applies learning-based methods to achieve mobility safety. Wan and Yamada [26] investigated the detailed gait analyses during walker-assisted walking. The changes in determinism of gait dynamics owing to the intervention of a robotic walker can be identified. Morone et al. [27] evaluated the effects of overground robotic walking training performed with the servo-assistive device, named *i*-Walker, on walking balance and gait stability in patients with mild subacute stroke. Ferrari et. al. [28] investigated the interaction between users and a robotic walker named *FriWalk*. The walker has the capability to navigate and guide the user through indoor environment along a planned path. In addition, a utility approach was proposed for a robot-assisted navigation, where user intent adjustments can be learned by reinforcement learning [29].

User’s intention is very useful for the applications of active walkers. With the user’s intension, smart walkers can provide power-assistance to help users walk safely and comfortably. In the study of user’s intention, both the vision-based and force sensing approaches can be found in the literature. In the vision-based approach, a camera is installed to detect the user’s movement intension [10,13,15]. On the other hand, some pressure or force sensors are mounted on the handle to detect the user’s posture [25,29,30]. For the movements like push and pull, the detection from force sensing will be more straightforward. Also, the installation of force sensors is more convenient and the cost is cheaper than the vision-based approaches. In the past, smart walkers were often restricted with some limitations. For example, the user needs to change the gait speed according to the street conditions such as slowing down the gait speed while walking through a ramp. The elderly may not have enough strength to control the walker brake by themselves. Ultimately, it leads to an increase in the risk of falling.

The proposed smart walker can automatically control the speed of the walker according to the surface slope and the user’s posture. In this study, the flexiforce sensor is used to detect the user’s posture and the user’s intention accordingly whether to move forward or backward. While using flexiforce sensors, small changes occurred in posture can be easily recognized from the difference between the sensors. In this paper, it is desired to design and implement an active-mode walker for the elderly. The motor will take the decision to move forward or backward based on the fuzzy inference rules to make the user’s walk smoother. The fuzzy inference system is easy to be implemented without knowing the plant models. In this study, the slope gradient, velocity of walker, grip forces values are considered as the input variables to generate a proper assistant force. Moreover, if there is an obstacle detected by the walker, the user will be alerted and the walker will be decelerated to ensure the safety of the user. Considering the cost effect and possible working environment, the ultrasonic sensor is applied for obstacle detection. The proposed system is built in a network-connected scheme, where the user status and surrounding environment data are sent to the cloud database. Finally, for the sake of safety monitoring, an App is developed that allows the families to know the user’s current status.

The object of this study is to develop an active walker that can provide power assistance to help the user walk safely and comfortably. According to the slope of a surface and velocity of the walker, an assistive power can be generated from a fuzzy inference scheme. Moreover, the user’s postures can be identified from the sensing grip forces. The nominal fuzzy rules will be remedied with the given user’s postures that will be more appropriate for the walker moves on flat, uphill, or downhill surfaces. The research subjects mainly include (1) how to identify the user’s postures with the grip forces, (2) how to fine tune the fuzzy rules according to the slope of surface, velocity of walker, and user’s postures, (3) experimental tests and results analyses to validate the effectiveness of the proposed walker. The peripheral sensors and computing kernel will be integrated together based on a ROS framework. The potential contributions of this study include (1) the user’s postures can be identified from grip forces with the benefits of easy installation and cost effectiveness, (2) a combined fuzzy inference scheme is proposed to provide power assistance that will be more appropriate for the elderly to prevent falling, (3) an App will be developed to help the user’s families remotely monitoring the user’s status that could increases the system effectiveness of the proposed walker.

## 2. Walker Design and Implementation

**Figure 1:**
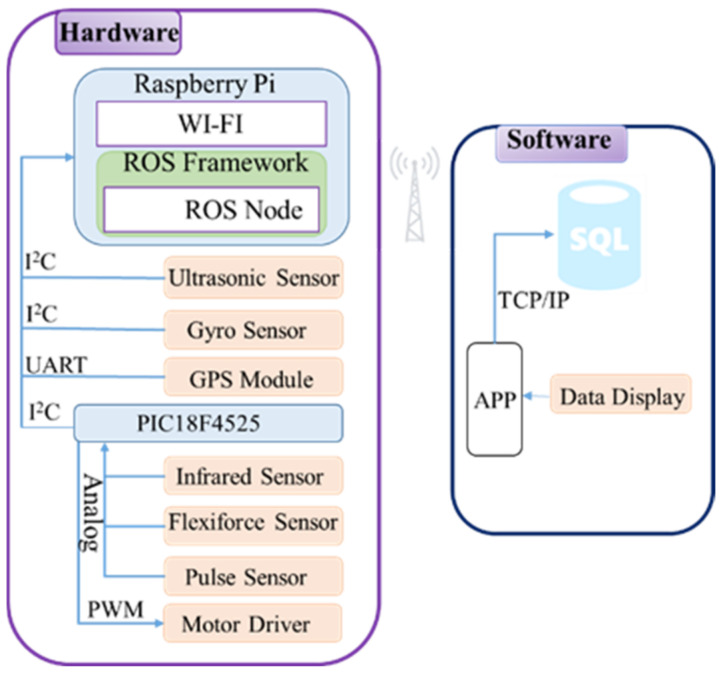
Proposed smart walker architecture.

The proposed system is divided into two parts, hardware and software, as shown in Figure 1. In the hardware part, the control kernel is a single-board computer Raspberry Pi combined with PIC microcontroller for sensing and motor control. Raspberry Pi and PIC microcontroller can work efficiently as the control kernel and provide many facilities [31,32]. In this study, Raspberry Pi 3 B+ (Adafruit, New York, NY, USA) equipped with ROS framework and PIC18F4525 (Microchip Technology Inc., Taipei, Taiwan) is used as the control core. The sensing data are collected by the PIC and transmitted to Raspberry Pi through the I2C protocol. SRF08 ultrasonic sensor (Active Robots Limited, Chilcompton, UK) is used for obstacle detection. SRF08 has a range of 3 cm to 6 m. Also, it has the capability of obstacle detection in front as well as in a conical shape 45 degrees. It generates frequency above 20 kHz, so it is not harmful to the human being as it is higher than the human audible range. Two Flexiforce A201(Tekscan, Boston, MA, USA) are used for measuring the force values exerted by the user. A standard A201 Flexiforce sensor is available in three ranges, 0–1, 0–25, and 0–100 (lbf). Here the one with 0–100 lbf has been used. MPU-6050 MEMS (InvenSense Inc., San Jose, CA, USA) motion tracking device, combining a 3-axis gyroscope and a 3-axis accelerometer, is used to measure the angular velocity. It can measure ±250, ±500, ±1000, ±2000 (dps), and the accelerometer can measure ±2, ±4, ±8, ±16 (g), so users can use it according to their needs. SEN-11574 pulse sensor (Hobbytronics limited, Wilberfoss, UK) is used to obtain the pulse rate. A NEO-6M GPS module (u-blox, Taipei, Taiwan) is also used to send the user’s location to App. Reflective optical type CNY70 IR sensor (Hobbytronics limited, Wilberfoss, UK) is used in the design of the rotary encoder to identify the motor rotation in forward or reverse direction and also the speed of the motor. The design of a rotary encoder is basically composed of two CNY70 IR sensors and one encoder disc. The rotary encoder is mainly used to measure the motor rotation speed and the forward/reverse rotation. In this paper, the encoder disc consists of 36 sets of black-and-white grids. The wheel size is 20 cm in diameter. Thus, when the infrared sensor detects a set of a black-and-white grid of the encoder disc, the moving distance of the wheel is about 1.7 cm. To determine the rotational direction, two sets of CNY70 sensors are used. Also, the VNH5019A-E motor driver (STMicroelectronics, El Paso, TX, USA) is used for PWM switching control.

**Figure 2:**
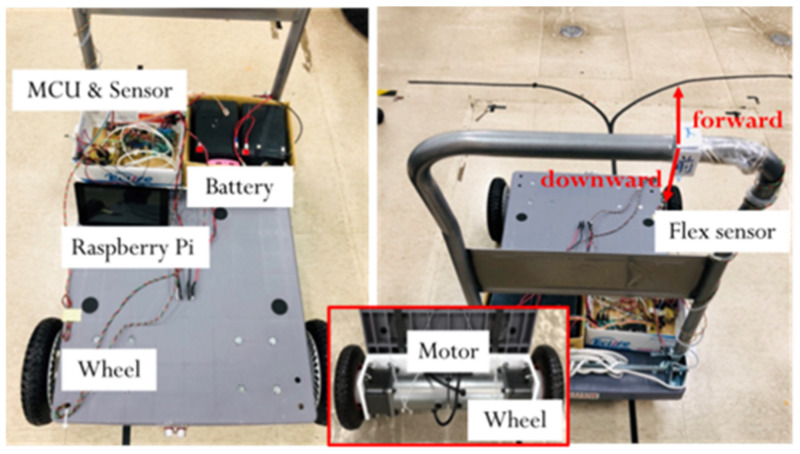
Real design of the proposed smart walker.

**Figure 3:**
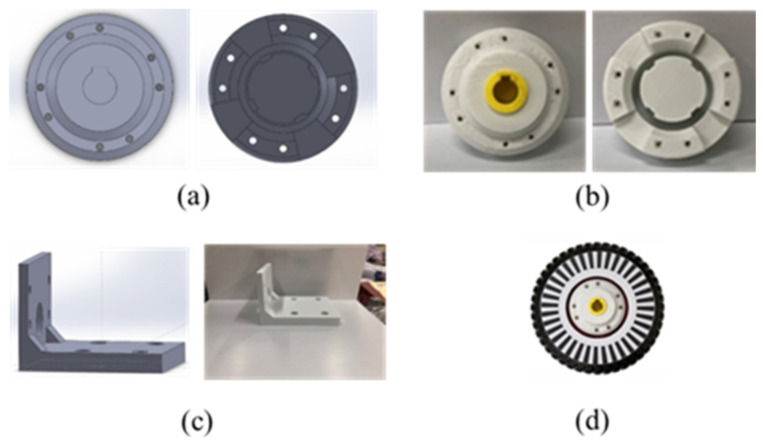
Real design of the proposed smart walker. (**a**) SolidWorks drawn coupling, (**b**) 3D printer made coupling, (**c**) L-shaped bracket with SolidWorks drawing and 3D printing, (**d**) wheel with coupling device and rotary encoder disc.

The developed smart walker is shown in Figure 2, where a pushcart is utilized as the frame structure such that the installing of sensors and active power-aided wheels are easily performed. In Figure 2, two flexiforce sensors are fixed on the handle to measure the grip strength downwards and forwards, respectively. Especially, two front castors of the cart are replaced with motor-driven wheels. Some other components are designed and made by SolidWorks and 3D printing, as shown in Figure 3. For example, a coupler has been made such that the motor and wheel can be tightly coupled, shown as Figure 3a,b. In addition, an L-shaped bracket was made in order to mount the motor on the walker, as shown in Figure 3c. The wheel with the coupling device along with the designed rotary encoder disc is shown in Figure 3d. The designed system allows the smart walker to judge the user’s posture and surrounding information and control the motor accordingly. The user can easily operate the necessary functionalities. In this study, the combination of a 12SGU-24V-3200R DC motor, 24 V 200 W, and a 5GX-50K speed reducer is considered. Due to the requirement of large torque at low speed, the 3200 rpm motor is matched with a 50:1 speed reducer. Two lead-acid batteries connected in series are used, 24 V 12 Ah. If the working duty is less than 50%, the battery can support the walker more than 90 min.

## 3. ROS-Based Fuzzy Controller Design with User’s Posture

**Figure 4:**
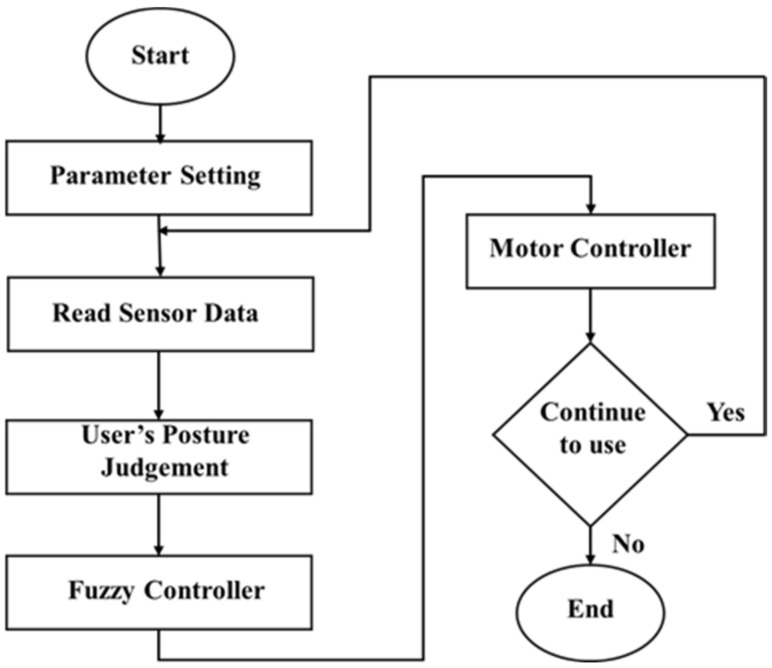
Execution flowchart of smart walker.

In the power-assistance design, a fuzzy controller is applied to make the manipulation more effective. The flow chart of the system execution process of the smart walker is shown in Figure 4. Particularly, both the surface situation and the user’s posture are taken into consideration. All the sensing data, including the surface slope, moving speed, and grip forces, are considered as the inputs to the fuzzy controller. Then the defuzzified output provides a decision as the demand to the motor. The details are discussed in the following Section.

### 3.1. ROS Framework

In the proposed system, the robot operating system (ROS) is used as a software framework. ROS is an open-source middleware, providing services like hardware abstraction, low-level device control, implementation of a commonly used function, message transmission between the nodes, and package management [33]. A node is a process that performs computation. ROS nodes use a ROS client library to communicate with other nodes. ROS provides a number of libraries for doing complex tasks such as running multiple sensors simultaneously. This means that sensor nodes can be executed independently at a time without affecting each other. In the ROS framework, the so-called message, first delivered to the topic, is transferred from one node to another node. The topic is similar to a bulletin board where nodes post their messages and each node can freely access. The node that sends a message is called Publisher, and the node that receives a message is called a Subscriber. The ROS-based framework is really flexible and adaptable to the needs of the user.

**Figure 5:**
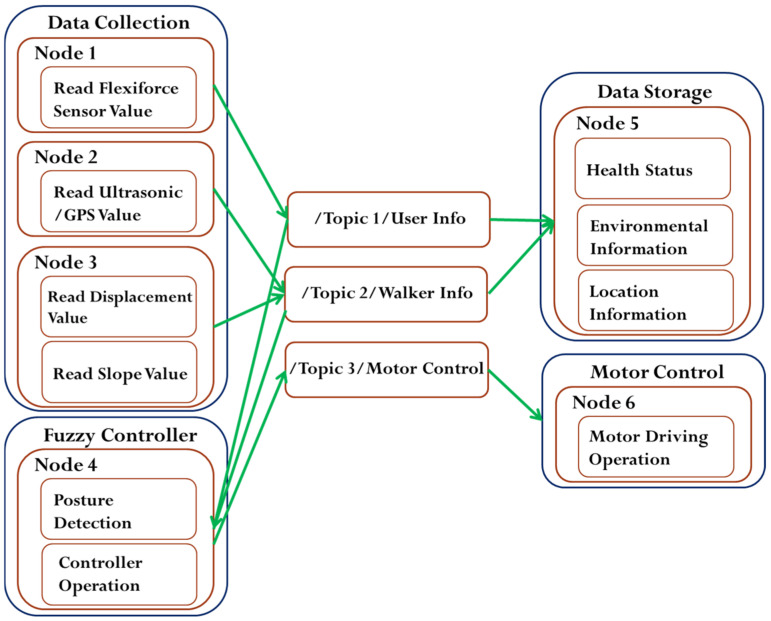
Robot operating system (ROS) framework of smart walker.

In this study, the system integration, including the data sensing and fuzzy controller design, is based on a ROS framework as shown in Figure 5. In this ROS framework, the whole system is divided into four packages, namely data collection, fuzzy controller, data storage, and motor control. In a package, each node transmits data among other nodes through topics by acting as publisher and/or subscriber. Taking a close look at the ROS framework, the fuzzy controller receives the data about the user’s posture and the surface slope and then provides an output decision which becomes an input to the motor controller. Under the ROS framework, each node can perform one-to-one, one-to-many, many-to-one, and many-to-many data sharing regardless of a publisher or a subscriber. The advantage of writing a program under the ROS framework is that the program execution of Node1~Node6 can be performed separately in a multiplexed manner. Thus, the complexity of program coding can be reduced and the program fault forbearance rate becomes higher. More importantly, under the ROS framework, the entire program will not be failed due to a single node error.

### 3.2. Fuzzy Controller Design

The readings of the gyro sensor, rotary encoder, and two flexiforce sensors are considered for the fuzzy controller design. The data from the gyro sensor can be used to determine whether the current road surface is rising upward, flat, or declining downward. The encoder reading indicates the movement status of the walker, such as moving forward, standing still, or moving backward. In addition, two flexiforce sensors are used to measure the forces exerted by the user’s grip strength forward and downward.

**Figure 6:**
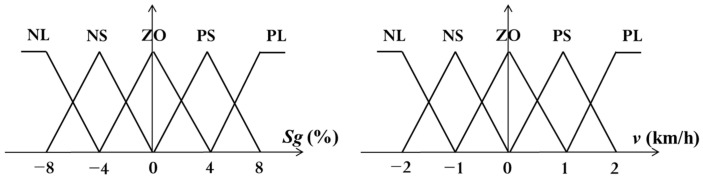
Input membership functions.

**Figure 7:**
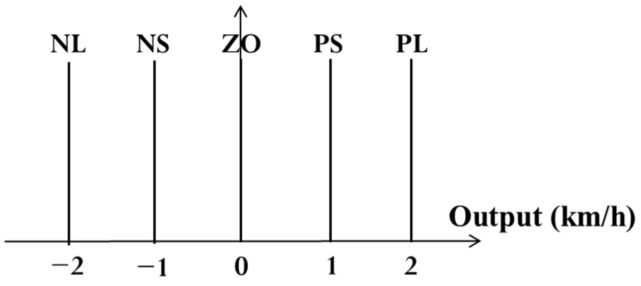
Output membership functions.

The Mamdani’s Min-Max inference method is used in this paper. First, the cases without the user postures are considered, where the slope gradient ($S_{g}$) and the moving speed (*v*) are the two input variables. The input membership functions are in triangular type, shown in Figure 6. The fuzzy if-then rules are illustrated in Table 1. The speed is considered as the output variable, where the membership function is in singleton type, shown in Figure 7. The linguistic variables of these fuzzy sets are NL (Negative Large), NS (Negative Small), ZO (Zero), PS (Positive Small), and PL (Positive Large). The design ideas of this study are described below in details. With the triangular input membership functions, the matching degrees of input data are easily obtained. Moreover, the output membership functions are singleton values such that the computational complexity of the defuzzification computation is significantly simplified. It is noticed that membership functions could be triangular, Gaussian, singleton, or other types. Basically, there is no restrictive rule for the selection of membership functions. The defuzzified outputs could be a little bit different due to selected membership functions. In real applications, the domain knowledge about the problem could be of much help, of which appropriate range of membership functions and fuzzy rules can be determined.

For the slope gradient $S_{g}$, Positive (P) means uphill and Negative (N) means downhill. For examples, PL means that the walker is moving uphill and the slope is greater than 4%, PS means the walker is moving uphill and the slope is between 0 and 8%, ZO means the walker is moving on a flat surface and the slope reading is between −4% and 4%. Similarly, NS means that the walker is moving downhill and the slope is between −8% and 0%. In addition, NL means the walker is moving downhill and the slope is less than −4%. For the speed *v*, Positive (P) means the walker is moving forward toward the user’s front direction, and Negative (N) means the walker is moving backward in the reverse direction. In Figure 5, PL and PS mean the walker is moving at more than 1 km/h and between 0 and 2 km/h, respectively, in forwarding direction, ZO means walker speed is between −1 and 1 km/h. Similarly, NS and NL mean the walker speed is −2~0 km/h and less than −1 km/h, respectively, in the reverse direction. In the output, Positive (P) means that an additive forward force will be produced along with the user’s front direction. In the same way, Negative (N) means that a reversal force will be generated to the walker toward the backward direction. For example, PL and PS mean that a forward force of 2 km/h and 1 km/h will be fed to motor as controller output respectively. Similarly, NL and NS mean that a reversal force of −2 km/h and −1 km/h will be fed to motor as controller output, respectively. ZO indicates no need to change in speed meaning that the walker will keep the movement in previous state.

Note that the if-then rules in Table 1 consider only the stationary cases, where the designated rules are used to hold the walker standstill regardless of the walker speed and surface slope. Some of the design rules are explained below to understand the design concepts more clearly. For example, in the case of

“If $S_{g}$ is PL and *v* is NL, Then the output is PL,”

Here, the walker is placed on a steep uphill ramp, but the walker is moving backward at a large speed. Under this circumstance, a large forward force is required to hold the walker in stationary. For another case,

“If $S_{g}$ is NS and *v* is PS, Then the output is NS,”

Here, the walker is placed on a small downhill slope, and the walker is moving forward with a small speed. So, here, a small reversal force is required to hold the walker standstill.

### 3.3. User’s Posture Judgement

This study adds the user’s posture judgement to the fuzzy controller. This part plays an important role for the smart walker because this walker not only helps the user in walking but also protects them from falling down while walking. Thus, two flexiforce sensors are placed on the handrail of the smart walker. The values of the forces exerted are considered to remedy the fuzzy rules. Both of the sensors reading can be used to analyze the user’s current posture. The force exerted by the two sensors is named as the forward force $f_{f}$ and downward force $f_{d}$. Again, both the forces are divided into large (L: >80 lbf), medium (M: 30~80 lbf), and small (S: <30 lbf). As three categories of forces, there are a total of nine possibilities for posture judgment. With the change of the reading values of $f_{f}$ and $f_{d}$ the current posture of the user can be identified, shown as in Table 2, Table 3 and Table 4. The cases in a flat surface are addressed in Table 2, and the cases of moving uphill and downhill are summarized in Table 3 and Table 4, respectively. This posture judgement will provide appropriate assistance to the users to walk comfortably and safely on a flat or ramp surface.

**Figure 8:**
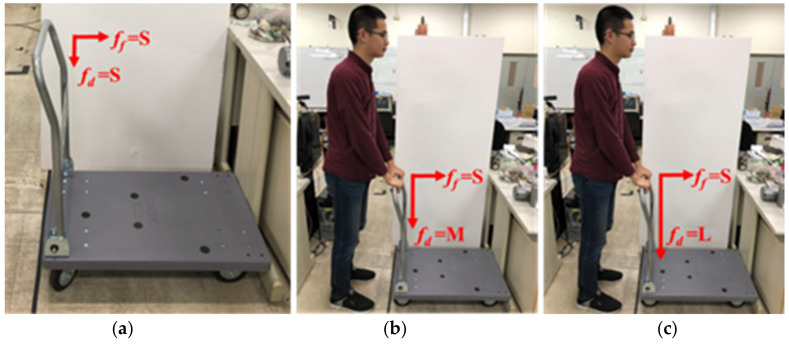
Posture judgment ($f_{f}$ = S): (**a**) $f_{d}$ = S; (**b**) $f_{d}$ = M; (**c**) $f_{d}$ = L.

**Figure 9:**
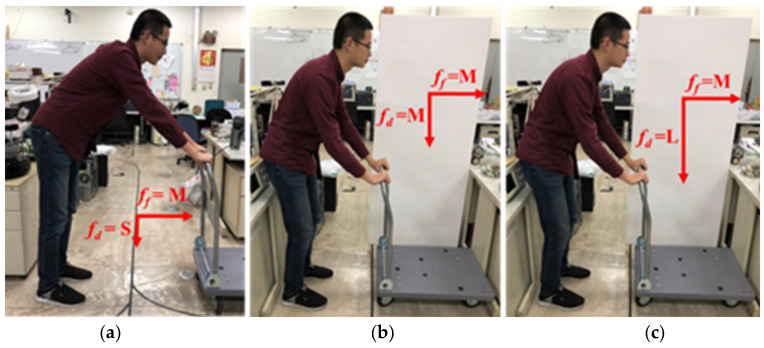
Posture judgment ($f_{f}$ = M): (**a**) $f_{d}$ = S; (**b**) $f_{d}$ = M; (**c**) $f_{d}$ = L.

**Figure 10:**
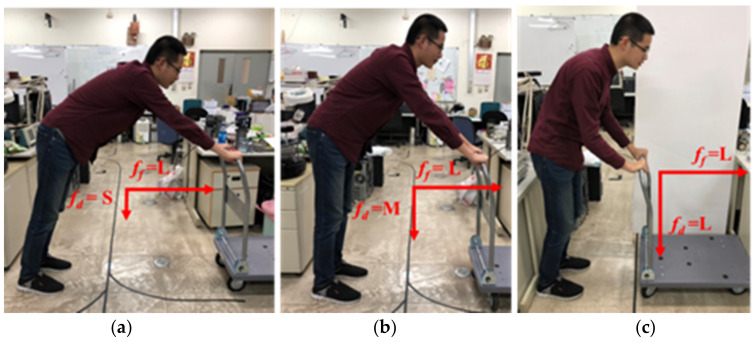
Posture judgment ($f_{f}$ = L): (**a**) $f_{d}$ = S; (**b**) $f_{d}$ = M; (**c**) $f_{d}$ = L.

The details about the postures in different surfaces are explained in the following. The postures on a flat surface are quite intuitive. To make the explanation of postures easier, the nine postures in flat surface are shown in Figure 8, Figure 9 and Figure 10. For example, if $f_{d}=L$ and $f_{f}=L$, the user is most likely struggling hard to push the walker as it could not move (c.n.m). In the case of $f_{d}=M$ and $f_{f}=L$, the user is leaning forward slightly (l.f.). Moreover, if $f_{d}=S$ and $f_{f}=L$, the user is likely bending forward (b.f.). Also, in Table 3, n.w. stands for normal walking, l.o. stands for lean on the walker, and s.s. represents stand still.

As the walker moving uphill, same force readings of $f_{d}$ and $f_{f}$ may indicate different postures in a few cases. For example, if $f_{d}=S$ and $f_{f}=M$, the user posture is bending forward on a flat surface. However, while moving uphill, the posture recognized as leaning forward is more appropriate. On the other hand, while the walker is moving downhill, most cases of the same reading of fore sensors indicate the same user postures like the uphill cases. Except that if $f_{d}=L$ and $f_{f}=L$, the user posture is more likely to be leaning forward.

### 3.4. Remedy of Fuzzy Rules

Table 1 gives the nominal fuzzy rules according to the surface slope and walker velocity. So far, the postures of the user are not involved. With the consideration of user postures, some of the fuzzy rules are required to be modified to provide comfort and safety to the users. Based on the implementation of the proposed smart walker, the user moves only in forward direction, thus the walker velocity *v* is greater than or equal to zero. Hence, only ZO, PS, and PL cases of *v* are investigated while the user’s postures are considered. In the following, two power-assistant design concepts are provided for the remedy of fuzzy rules, as shown in Algorithms 1 and 2. The notations ⋁ and ⋀ stand for the logic OR and AND, respectively. From the discussion in Section 3.3, user’s postures can be identified from $f_{f}$ and $f_{d}$, and all possible postures can be categorized as normal walking, lean forward, and bending forward, etc. With user’s postures, the adjustment of the fuzzy rules will be discussed in the following. As the $f_{f}$ and $f_{d}$ are divided into three categories L, M, and S, there are nine remedy fuzzy tables, out of which three tables are shown as examples in Table 5, Table 6 and Table 7.

First, the cases of *v* = ZO are addressed, and the design concepts are summarized in Algorithm 1. From Table 1, if the slope $S_{g}$ = PS, the corresponding controller output is PS without the consideration of the postures. Moreover, if $f_{f}$ = L and $f_{d}$ = S, the user is bending forward from Table 4. In this situation, the walker needs to slow down, thus the corresponding controller output is modified to NS for fall prevention. Similarly, originally if $S_{g}$ = ZO, the controller output is ZO in Table 1. But with the posture $f_{f}$ = L and $f_{d}$ = S, the controller output is changed to NS in order to maintain safe operation. Considering the posture $f_{f}$ = L and $f_{d}$ = S, the remaining cases of different $S_{g}$ and *v* are analyzed in the same way, and the adjustments are summarized in Table 5.

Then the cases of *v* = PS are discussed, and the design concepts are summarized in Algorithm 2. In a flat surface, $S_{g}$ = ZO, the original controller output is NS from Table 1. But, with $f_{f}$ = L and $f_{d}$ = L, it implies that the user is pushing hard to move the walker. So, a slightly forward force is required for the movement of walker. Thus, the controller output is changed to ZO as shown in Table 6. Similarly, if $S_{g}$ = PS, the controller demand is ZO without the consideration of postures form Table 1. Since the user is pushing hard to move uphill, more forward force is required, and the controller output is changed to PS, as shown Table 6.
**Algorithm 1:** Power assistance with user’s posture (*v* = ZO)Input variables: $S_{g}$, $f_{f}$, $f_{d}$, *v*
While $S_{g}$ = PS or PL If $f_{f}$ = (L ⋁ M) ⋀ $f_{d}$ = S, then controller output = *slower or reversal*  else controller output = *forward* While $S_{g}$ = ZO If ($f_{f}$ = (L ⋁ M) ⋀ $f_{d}$ = S) ⋁ ($f_{f}$ = L ⋀ $f_{d}$ = M), then  controller output = *slow reversal* else if ($f_{f}$ = (L ⋁ M) ⋀ $f_{d}$ = L) ⋁ ($f_{f}$ = M ⋀ $f_{d}$ = M), then controller output = *forward slowly* else *stay the same* While $S_{g}$ = NS or NL  If ($f_{f}$ = M) ⋀ ($f_{d}$ = L ⋁ M), then controller output = *slower than general*
 else controller output = *reverse* (*fast* or *slow*) End**Algorithm 2:** Power assistance design user’s posture (*v* = PS or PL)Input variables: $S_{g}$, $f_{f}$, $f_{d}$, *v* While $S_{g}$ = PS or PL If ($f_{f}$ = L ⋀ $f_{d}$ = L) ⋁ ($f_{f}$ = M ⋀ $f_{d}$ = L) ⋁ ($f_{f}$ = M ⋀ $f_{d}$ = M), then   controller output = *forward fast* else if ($f_{f}$ = L ⋀ $f_{d}$ = M) ⋁ ($f_{f}$ = L ⋀ $f_{d}$ = S) ⋁ ($f_{f}$ = M ⋀ $f_{d}$ = S), then controller output = *reverse* (*slow* or *fast*)  else *stay the same* While $S_{g}$ = ZO  If $f_{f}$ = L ⋀ $f_{d}$ = L, then  controller output = *forward fast* else if $f_{f}$ = M ⋀ $f_{d}$ = (L ⋁ M), then controller output = *stay the same*
 else *reverse* (*slow* or *fast*) While $S_{g}$ = NS or NL  controller output = *reverse slow* (*v* = PS) or *reverse fast* (*v* = PL) End

## 4. Experimental Results and Analysis

### 4.1. Design of Experiments

**Figure 11:**
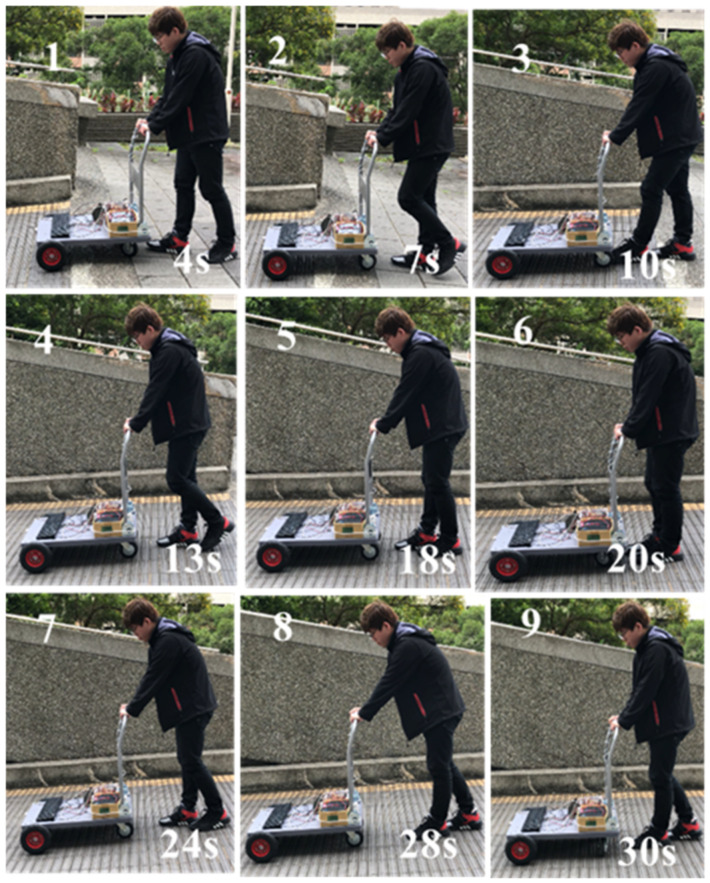
Snapshots of smart walker assisted in downhill.

**Figure 12:**
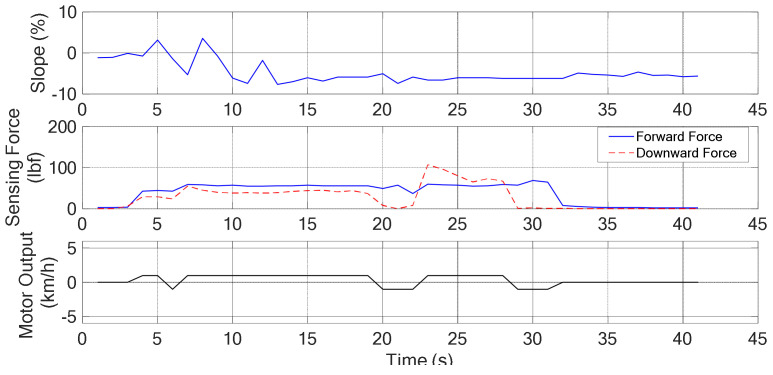
The degree of slope, sensing force, and the assistive motor output (downhill).

**Figure 13:**
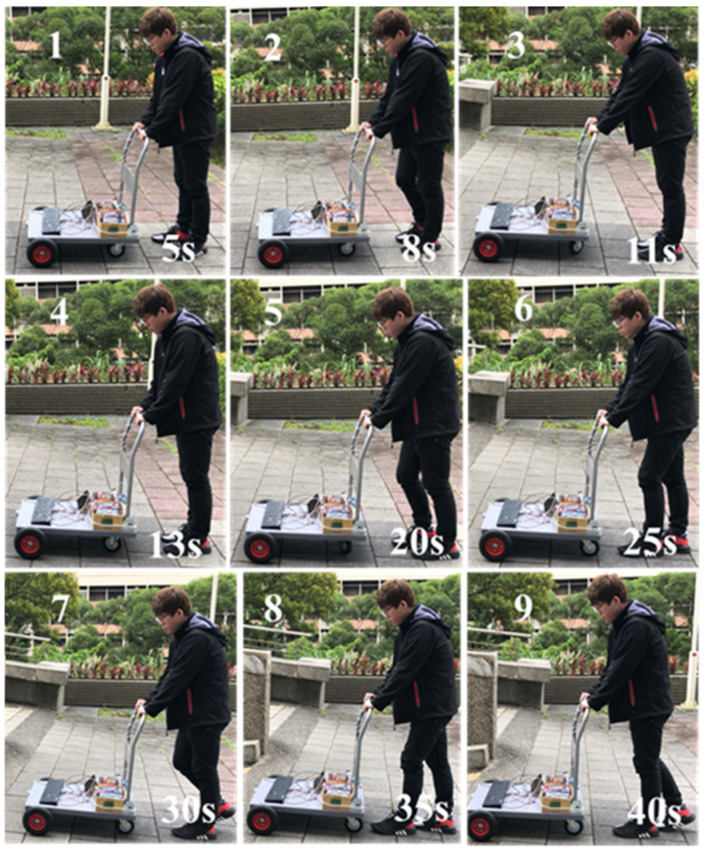
Snapshots of smart walker assisted in flat surface.

**Figure 14:**
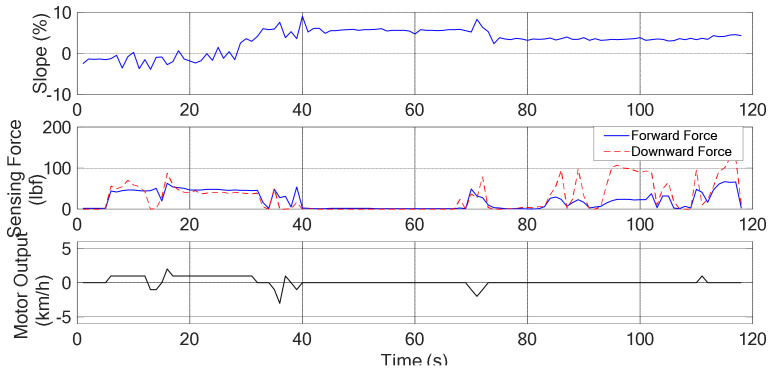
The degree of slope, sensing force, and the assistive motor output (flat surface).

**Figure 15:**
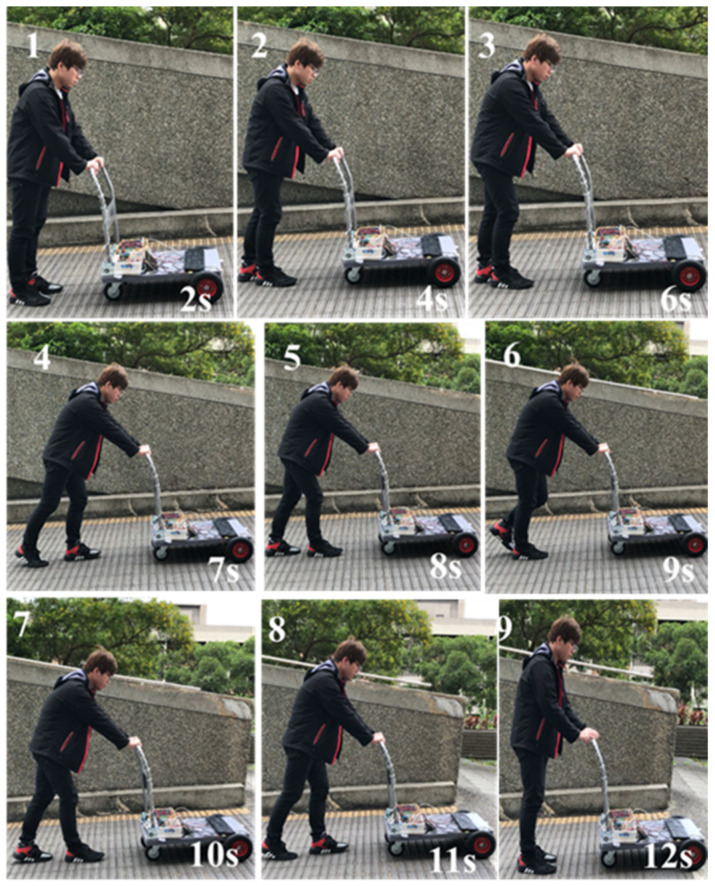
Snapshots of smart walker assisted on a steep uphill.

**Figure 16:**
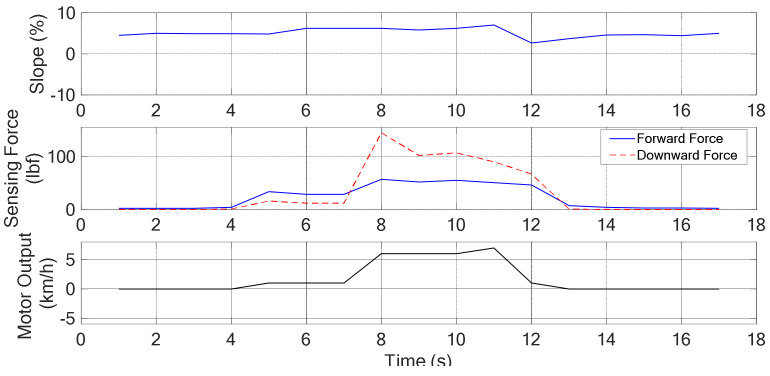
The degree of slope, sensing force, and the assistive motor output (steeper uphill).

The proposed system experimentation and the usage scenario of the smart walker are described in detail. The controller input and output value comparison are presented as shown in below graphs. In each graph the slope gradient is defined as −10% to 10% (Negative sign: downhill, Positive sign: uphill), the sensed grip force values are divided into large, medium, and small and the range is set between 0 and 150 lbf. The output is the motor output and the range is set between −5 km/h and 5 km/h (Negative sign: reverse force, Positive sign: forward force). For the posture judgment and to verify whether the designed fuzzy control is reasonable or not we considered many circumstances with different slope and force readings. Here, the user resembles to an elderly people who has a slower walking speed. So, here the speed of the walker is considered as ZO (−1 km/h to 1 km/h). As, the walker is considered to move in front direction only, so here ZO means the moving speed is less or equal to 1 km/h. Now, the designed system is experimented for real-time with three different slope gradients and the obtained results are explained below in detail. The snapshots are taken to show the user’s postures while walking on different slopes as shown in Figures 11, 13, and 15. In the following cases, the control output corresponding to the slope gradients and the grip forces are shown in Figures 12, 14, and 16, respectively. The parameter settings of the fuzzy power-assistance and posture judgements are summarized in Table 8. The following experimental tests and results analyses are carried out. The arrangements of experimental results corresponding to different environments are also indicated in Table 8. The information about the participants who are involved in the function modules or integration tests are listed in Table 9.

### 4.2. Results and Analyses

#### 4.2.1. Moving in a Downhill Surface

The snapshots are shown in Figure 11 and the corresponding recorded data are shown in Figure 12. In the 1st sub photo of Figure 11, it is shown that the user moves from flat surface toward downhill. Thus, the obtained slope graph ranges from 0% to −7% as shown in Figure 12. It is noted that the obtained graphs are not smooth due to the surface tiles pattern. In the 4th sub photo of Figure 11, here $S_{g}$ = NS, $f_{f}$ = M, $f_{d}$ = M, and *v* = ZO, that can be seen in Figure 12 at 13 s. From Table 2, without considering the posture, if *v* = ZO and $S_{g}$ = NS, then controller output = NS. But, from the sensing forces, the status $f_{f}$ = M and $f_{d}$ = M indicate the “normal walking” posture as shown in Table 5. It means that the user wants to walk forward with a normal speed. So, with the addition of posture, the controller output is changed from NS to ZO, as shown in Table 8. Hence the motor continued to produce speed +1 km/h for normal walking as shown in Figure 12. Then the 5th and 8th sub photos are considered. The sensing forces $f_{f}$ = M and $f_{d}$ = S indicate “lean forward” as shown in Table 5. In this situation, the walker is gradually moved away from the user, and the user may have chances of falling. Thus, the walker is required to slow down, so that the user can gradually regain the center of gravity and return to the normal posture of walking. From Algorithm 1, under this circumstance, the fuzzy controller output is changed from NS to NL. Consequently, a reverse force of −1 km/h is generated as shown in Figure 12 at 18 s and 28 s, respectively. After the walker is moved back near to the user, shown in the 6th sub photo of Figure 11, the $f_{d}$ is gradually increased to M for normal walking, as the 7th sub photo of Figure 11. Previous explanations are summarized in Table 10.

#### 4.2.2. Moving on Flat Surface

The snapshots are shown in Figure 13 and the corresponding recorded data are shown in Figure 14. Here, $S_{g}$ = ZO and *v* = ZO. From Table 1, the fuzzy controller output is ZO without considering the posture. Considering the 1st sub photo of Figure 13, it can be seen that $f_{f}$ = M and $f_{d}$ = M from the sensing graph of Figure 14. This indicates “normal walking” posture as mentioned in Table 2. With the addition of the user’s postures, the fuzzy controller output is changed to PS as a forward force is required to move the walker as shown in Table 7. Consequently, a forward force of 1 km/h is generated as shown in Figure 14 at 5 s. In addition, the 3rd sub photo of Figure 13 is considered, where the sensing forces, $f_{f}$ = M and $f_{d}$ = S, can be observed in the sensing graph of Figure 14. This situation indicates the posture “lean forward” as mentioned in Table 2. Under this circumstance, the walker needs to move in reverse direction near to the user, so that the user can regain the center of gravity and continue normal walking. From Algorithm 1, the fuzzy controller output will be changed from ZO to NS, and a reversal force of −1 km/h is generated as shown in Figure 14 at 11 s. Moreover, $f_{f}$ = M and $f_{d}$ = M during 20~25 s, indicating that the posture is in normal walking status, thus the driving force stay the same as desired. Previous explanations are summarized in Table 11.

#### 4.2.3. Moving on a Uphill Surface

The snapshots are shown in Figure 15 and the corresponding recorded data are shown in Figure 16. In between the 2nd and 3rd sub photos, $S_{g}$ = PS, *v* = ZO, $f_{f}$ = S, $f_{d}$ = S, it can be observed that the walker moves from the standstill to normal walking. Thus, a forward force is generated as expected. Starting from the 7th second, it can be seen that the grip forces are increased, $f_{f}$ = M, $f_{d}$ = L. From Table 5, it indicated that the walker is in the status of normal walking. From Algorithm 1, more forward force is required to keep normal walking while the slope $S_{g}$ = PS or PL. These are accorded to the experiment results shown in Figure 16. Previous explanations are summarized in Table 12.

**Figure 17:**
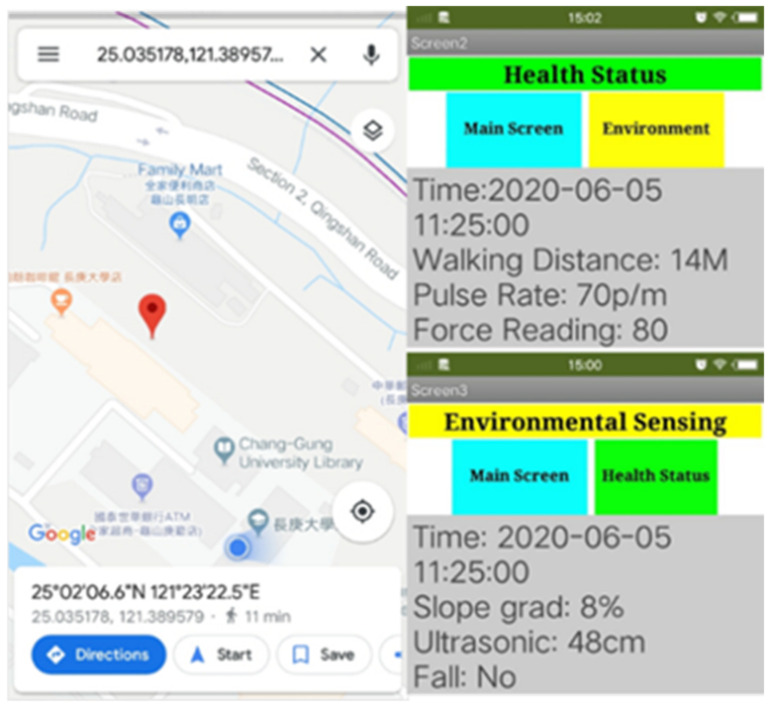
App showing: User’s location, user’s health status and environmental information encountered by smart walker.

The software part contains database and mobile APP. For database implementation MySQL database management system is used which is free and open source platform by Apache Friends. Here, the SQL, PHP, and JavaScript programming languages were used. All the data that are sensed by the sensors are stored in the database for future purpose. So, an android application is developed that can be used remotely to access the data from the database. The App contains the information about walking distance, pulse rate, slope, current user posture and current location of the user. In the first page of the App, it shows the overall information of the user and also the location as shown in Figure 17. Using this latitude and longitude, the location of the user can be found. In this case, the coordinates shown in App is near the Engineering Building in Chang Gung University. Furthermore, if we click Health Status and Environment tabs, it will show more information as shown in Figure 17.

## 5. Conclusions and Future Work

In this paper, we have presented an active smart walker that could help the elderly as well as to the people who need support to walk independently and safely. The device has the functions of intelligent control, posture judgment, environment sensing, and real-time monitoring. From the grip forces, six postures can be identified. Three scenarios, flat, downhill, and uphill surfaces, are considered for the experimental testing. The user can get power-assistance in walking and can also be protected from collision with obstacle. If the user has a possibility of falling, the motor can immediately control the walker to stabilize the user’s posture. In addition, an App has designed, so that family members or doctors can instantly get the current status of the user. If the user encountered accident like falling or losing balance, then this information will be updated to the database and the same information can be obtained through the App. Thus, the proposed walker not only helps in assisting, but also includes the scope of care for elderly. In the future, machine learning algorithms can be considered to enhance the values of this proposed walker. For example, the deep learning algorithms will be integrated with the lower limb posture recognition. The user’s recovery situations can be recorded and analyzed from the data like walking speed and walking pattern. The analysis results could help doctors to judge the treatment procedures to improve the patient’s recovery.

## Figures and Tables

**Table 1:** Fuzzy rule base (without user’s posture).

Controller Output	$S_{g}$					
*v*		PL	PS	ZO	NS	NL
	NL	PL	PS	PL	NS	ZO
	NS	PL	PS	PS	ZO	NL
	ZO	PL	PS	ZO	NS	NL
	PS	PL	ZO	NS	NS	NL
	PL	ZO	PS	NL	NS	NL

**Table 2:** Posture while in flat surface.

Posture		$f_{d}$		
		L	M	S
$f_{f}$	L	c.n.m	l.f.	b.f.
	M	n.w.	n.w.	b.f.
	S	l.o.	l.o.	s.s.

**Table 3:** Posture while moving uphill.

Posture		$f_{d}$		
		L	M	S
$f_{f}$	L	c.n.m	l.f.	b.f.
	M	n.w.	n.w.	l.f.
	S	l.o.	l.o.	s.s.

**Table 4:** Posture while moving downhill.

Posture		$f_{d}$		
		L	M	S
$f_{f}$	L	l.f.	l.f.	b.f.
	M	n.w.	n.w.	l.f.
	S	l.o.	l.o.	s.s.

**Table 5:** Fuzzy rule base (with user’s posture; $f_{f}$ = L, $f_{d}$ = S).

Controller Output	$S_{g}$					
*v*		PL	PS	ZO	NS	NL
	ZO	NL	NS	NS	NS	NL
	PS	PS	NS	NL	NL	NL
	PL	PS	ZO	NL	NL	NL

**Table 6:** Fuzzy rule base (with user’s posture; $f_{f}$ = L, $f_{d}$ = L).

Controller Output	$S_{g}$					
*v*		PL	PS	ZO	NS	NL
	ZO	PL	PL	PS	ZO	NS
	PS	PL	PL	ZO	ZO	NS
	PL	ZO	ZO	ZO	ZO	NS

**Table 7:** Fuzzy rule base (with user’s posture; $f_{f}$ = M, $f_{d}$ = M).

Controller Output	$S_{g}$					
*v*		PL	PS	ZO	NS	NL
	ZO	PL	PL	PS	ZO	NS
	PS	PL	PS	ZO	ZO	NS
	PL	ZO	ZO	NS	ZO	NS

**Table 8:** Parameter settings and experimental results.

Fuzzy Controller	
Slope $S_{g}$	setting as Figure 6
Velocity v	
Output	setting as Figure 7
**Posture judgment**	
Grip force $f_{f}$, $f_{d}$	L: >80 lbf; M: 30~80 lbf; S: <30 lbf
**Experimental results**	
Downhill	shown as Figure 11, Figure 12
Flat surface	shown as Figure 13, Figure 14
Uphill	shown as Figure 15, Figure 16

**Table 9:** Participants involved in testing.

	$T_{1}$	$T_{2}$	$T_{3}$	$T_{4}$	$T_{5}$	$T_{6}$	$T_{7}$	$T_{8}$	$T_{9}$	$T_{10}$	$T_{11}$	$T_{12}$
Age	25	26	24	27	23	25	27	25	23	23	51	61
Gender	M	M	M	F	M	M	M	F	M	M	M	M
Height (cm)	174	183	165	155	174	172	163	159	170	169	176	175
Weight (kg)	70	65	75	60	83	71	52	53	60	83	85	86

**Table 10:** Summary of experimental results (Downhill: *v* = ZO, $S_{g}$ = NS).

Timestamp	Sub plot	Grip force	Posture	Control remedy
13 s	4th	$f_{f}$ = M, $f_{d}$ = M	Normal walking	NS → ZO
*Analysis*: Keep the same state to maintain “normal walking” *Afterwards*: Walker keep moving forward with a normal speed (13 s~18 s) *Evaluation*: Meet expectations or not. Answer: YES				
Timestamp	Sub plot	Grip force	Posture	Control remedy
18 s	5th	$f_{f}$ = M, $f_{d}$ = S	Leaning forward	NS → NL
*Analysis*: Need more backward force to regain stability *Afterwards*: Walker slows down and moves near to the user (18 s~20 s) *Evaluation*: Meet expectations or not. Answer: YES				

**Table 11:** Summary of experimental results (Flat surface: *v* = ZO, $S_{g}$ = ZO).

Timestamp	Sub plot	Grip force	Posture	Control remedy
5 s	1st	$f_{f}$ = M, $f_{d}$ = M	Normal walking	ZO → PS
*Analysis*: Need some forward force to main “normal walking” *Afterwards*: Walker moves forward with a normal speed (5 s~8 s) *Evaluation*: Meet expectations or not. Answer: YES				
Timestamp	Sub plot	Grip force	Posture	Control remedy
11 s	3rd	$f_{f}$ = M, $f_{d}$= S	Leaning forward	ZO → NS
*Analysis*: Need some backward force to regain stability *Afterwards*: Walker moves forward with a normal speed (13 s~25 s) *Evaluation*: Meet expectations or not. Answer: YES				

**Table 12:** Summary of experimental results (Uphill *v* = ZO, $S_{g}$ = PS).

Timestamp	Sub plot	Grip force	Posture	Control remedy
2 s~3 s	1st, 2nd	$f_{f}$ = S, $f_{d}$ = S	Standstill → n.w.	ZO → PS
*Analysis*: Need some forward force to attain “normal walking” *Afterwards*: Walker moves forward with a normal speed (4 s~7 s) *Evaluation*: Meet expectations or not. Answer: YES				
Timestamp	Sub plot	Grip force	Posture	Control remedy
7 s	4th	$f_{f}$ = M, $f_{d}$ = L	Normal walking	NS → NL
*Analysis*: More forward force is required to maintain normal walking *Afterwards:* Walker moves forward with a normal speed (8 s~10 s) *Evaluation*: Meet expectations or not. Answer: YES				
